# Thiamet-G facilitates reparative dentin formation via modulating O-GlcNAcylation and inflammation

**DOI:** 10.3389/fphys.2025.1739168

**Published:** 2026-01-16

**Authors:** Elina Pokharel, Tae-Young Kim, Bandana Rana, Je-Hee Jang, Jae-Hee Lee, Seo-Young An, Chang-Hyeon An, Hitoshi Yamamoto, Mee-Seon Kim, Wern-Joo Sohn, Youngkyun Lee, Jung-Hong Ha, Do-Yeon Kim, Jae-Kwang Jung, Jae-Young Kim

**Affiliations:** 1 Department of Biochemistry, School of Dentistry, IHBR, Kyungpook National University, Daegu, Republic of Korea; 2 Department of Anatomy, Keimyung University School of Medicine, Daegu, Republic of Korea; 3 Department of Oral and Maxillofacial Radiology, Kyungpook National University School of Dentistry, IHBR, ITRD, Daegu, Republic of Korea; 4 Department of Histology and Developmental Biology, Tokyo Dental College, Tokyo, Japan; 5 Department of Pathology, School of Dentistry, Kyungpook National University, Kyungpook National University Hospital, Daegu, Republic of Korea; 6 College of K-Biohealth, Daegu Haany University, Gyeongsan, Republic of Korea; 7 Department of Conservative Dentistry, School of Dentistry, IHBR, Kyungpook National University, Daegu, Republic of Korea; 8 Craniofacial Nerve-Bone Network Research Center, Kyungpook National University, Daegu, Republic of Korea; 9 Department of Pharmacology, School of Dentistry, IHBR, Kyungpook National University, Daegu, Republic of Korea; 10 Department of Oral Medicine, School of Dentistry, IHBR, Kyungpook National University, Daegu, Republic of Korea

**Keywords:** inflammation, OGA, O-GlcNAcylation, pulp cavity, reparative dentine, Thiamet-G

## Abstract

**Introduction:**

O-GlcNAcylation, a reversible post-translational modification regulated by O-GlcNAc transferase (OGT) and O-GlcNAcase (OGA), is involved in various cellular processes, such as proliferation, differentiation, and inflammation modulation. Developmental study revealed that proper O-GlcNAcylation mediated by OGT is vital for tooth morphogenesis. However, the function of O-GlcNAcylation during reparative dentin formation is still unknown. To understand its therapeutic relevance in regenerative dentistry, we examined the potential of OGA inhibitor, Thiamet-G, in reparative dentin formation using both *in vitro* and *in vivo* approaches.

**Methods:**

Human dental pulp stem cells were cultivated to examine cell viability, alkaline phosphatase (ALP) activity, and mRNA expression of reparative dentin-related genes. Furthermore, the dental pulp of the upper first molar in 8-week-old male ICR mice was exposed, and Thiamet-G was locally delivered for *in vivo* studies. Histological and immunohistochemical alterations were analyzed after 3 and 5 days post-cavity preparation, and dentin-bridge formation was evaluated at 42 days using histology and micro-CT.

**Results:**

*In vitro,* Thiamet-G treatment facilitated proliferation, ALP activity, and upregulated expression of reparative dentin-related genes, including BMP2, BSP, DSPP, OCN, and RUNX2. *In vivo,* Thiamet-G treated specimens showed the altered localizations of NESTIN, NF-κB, MPO, OPN, RUNX2, TGF-β1, and TNF-α at 3 and 5 days post exposure, suggesting enhanced dentin regeneration and modulated inflammation. Particularly, at 42 days, Thiamet-G treated specimens exhibited enhanced dentin-bridge formation, confirmed by micro-CT imaging and histology.

**Conclusion:**

Thiamet-G treatment facilitated reparative dentin formation by modulating inflammation and regulating regenerating signaling, suggesting its potential as a therapeutic agent.

## 1 Introduction

Odontoblasts are specialized mesenchymal cells which secrete dentin, a hard mineralized tissue beneath the enamel that protects the dental pulp. However, enamel damage caused by caries or trauma may expose the dentin, increasing the predisposition to pulpal injury and infection. In response, the odontoblast and progenitor cells initiate a process to differentiate into tertiary dentin, as a reparative mechanism (Jung et al., 2019). Unfortunately, this natural process of dentin repair is slow and often insufficient to restore the damaged tooth. Clinically, the exposed pulp tissue is covered with artificial mineral aggregates after devitalizing the tooth by removing the decay or trauma; but physiologic dentin regeneration still remains difficult to achieve (Moussa and Aparicio, 2019). Moreover, infection and inflammation further impede the repair and ultimately causing pulp necrosis and tooth loss (Cooper et al., 2014), highlighting the need for the therapies that support both regeneration and immune balance.

O-GlcNAcylation is one of the post-translation modifications in which a single sugar O-linked β-D-N-acetylglucosamine (O-GlcNAc) is added to the intracellular protein’s Ser/Thr residues (Torres and Hart, 1984). The addition and removal of O-GlcNAc is catalyzed by two enzymes: O-GlcNAc transferase (OGT) and O-GlcNAcase (OGA), respectively (Torres and Hart, 1984; Yang and Qian, 2017). This modification is essential in mammalian tissue specification, cell survival, and embryonic development (Yang and Qian, 2017; Shafi et al., 2000; Yang et al., 2012). Furthermore, O-GlcNAc levels vary in a tissue-specific manner during embryogenesis, human and murine mesenchymal stem cell-derived osteoblast differentiation, adipocyte, myoblast, and chondrocyte lineages (Yan et al., 2024; Ishihara et al., 2010; Andrés-Bergós et al., 2012; Czajewski and van Aalten, 2023). Several studies have indicated that O-GlcNAcylation plays a role in differentiating bone-forming cells, such as chondrocytes and osteoblasts (Yan et al., 2024; Nagel and Ball, 2014). For instance, increased O-GlcNAcylation was observed during osteoblastic differentiation of MC3T3 cells (Koyama and Kamemura, 2015). Moreover, OGA inhibition by Thiamet-G escalated Runt-related transcription factor 2 (RUNX-2) O-GlcNAcylation, thereby promoting osteogenic differentiation of BMSCs (Zhang et al., 2023; Koyama and Kamemura, 2015; Li et al., 2022). OGA inhibitors, such as PUGNAc and Thiamet-G, inhibited osteoclast differentiation with enhanced O-GlcNAcylation (Koyama and Kamemura, 2015; Li et al., 2022). These findings underscore the importance of precise regulation of O-GlcNAcylation in determining stem cell fate. Developmental study also demonstrated that OGT inhibition by OSMI-1 treatment during the cap stage of tooth development results in smaller teeth with fewer cusps, indicating that modulation of O-GlcNAcylation would regulate the formation of dental hard tissues, including dentin and enamel (Pokharel et al., 2023).

Thiamet-G, a potent and selective OGA inhibitor, has been widely used to investigate the role of O-GlcNAcylation in various biological contexts, including BMSC differentiation, neurodegenerative disorders, and other diseases (Yan et al., 2024). In cardiovascular diseases, O-GlcNAcylation upregulation through Thiamet-G treatment showed anti-inflammatory and vasculoprotective effects (He et al., 2017). Although O-GlcNAcylation is crucial in cell physiology and disease, its specific role in dentin regeneration still remains unexplored. Given its importance in other tissue regeneration and inflammation, further studies are essential to reveal its potential in dental tissue repair and regeneration. This study aimed to examine the molecular and cellular functions of O-GlcNAcylation in reparative dentin formation using Thiamet-G in both *in vitro* and *in vivo* models, offering new insights into therapeutic strategies for promoting natural tooth repair.

## 2 Materials and methods

### 2.1 Human dental pulp stem cells (hDPSCs) and Thiamet-G treatment

The hDPSCs purchased from Lonza (PT-5025, Lonza Bioscience) were cultured in DPSC SingleQuot Growth Medium (DPSCGM) (PT-4516, Lonza Bioscience) in a humidified atmosphere with 5% CO_2_ at 37 °C. For osteogenic differentiation, hDPSCs at passage 4 or 5 were seeded at a density of 2 × 10^4^ cells per well in 24 well collagen-coated plates. After 24 h, the media was changed with osteogenic media (Alfa-MEM with 1% penicillin-streptomycin, 5% fetal bovine serum, 50 μM L-ascorbic acid, 10 mM β-glycerophosphate, and 100 nM dexamethasone) in presence or absence of Thiamet-G (S7213, Selleckchem.com) with various concentrations. The culture media was changed every 2 or 3 days.

### 2.2 Cell viability assay

Human dental pulp stem cells (hDPSCs) viability was determined using the MTS assay. hDPSCs were seeded into 96-well plates at 5 × 10^3^ cells/well density in a serum-free medium and incubated in a humidified atmosphere with 5% CO_2_ at 37 °C. The next day, cells were treated with vehicle (DMSO) or Thiamet-G (1 μM, 10 μM, 50 μM, & 100 μM) in DPSCGM and further incubated at 37 °C for 24 h and 48 h. After the indicated drug treatment period, 20 μL MTS (G3582, Promega) solution was put into each well and incubated at 37 °C for an hour. The absorbance was examined using a SpectraMax ABS Microplate Reader at a wavelength of 490 nm. Cell viability was calculated using Excel.

### 2.3 Alkaline phosphatase (ALP) activity assay

hDPSCs at passage 4 or 5 were cultured with vehicle (DMSO) or Thiamet-G (1 and 10 μM) in osteogenic differentiation media for 7 and 14 days. To detect the osteogenic differentiation of hDPSCs, alkaline phosphatase (ALP) activity was performed using an ALP activity assay kit (ab83369, Abcam), according to the manufacturer’s protocol. The absorbance was measured at 405 nm using a SpectraMax ABS Microplate Reader. The standard curve and ALP activity were calculated using Excel, following the manufacturer’s instructions.

### 2.4 RNA extraction and real-time qPCR

hDPSCs at passage 4 were cultured in osteogenic media with vehicle (DMSO) or Thiamet-G (10 μM) for 7 days. Then, RNA was extracted using RNeasy® Micro Kit (74004; Qiagen) and transcribed to cDNA using Omniscript RT kit (205111; Qiagen), following the instructions in the manual. RT-qPCR was performed using the StepOnePlus RT-PCR system device. The 2^−ΔΔCT^ method was used to determine the relative alteration in gene expression, and glyceraldehyde-3-phosphate dehydrogenase (GAPDH) was used as an internal control. The nucleotide sequences of the primers used in this study are listed in Supplementary Table 1. The obtained data was analyzed using Excel and GraphPad Prism 8.

### 2.5 Animals

All experiments were approved by Kyungpook National University School of Dentistry, Intramural Animal Use and Care Committee (KNU 2020-0107). For this study, 8-week-old male Institute of Cancer Research (ICR) mice were used for pulp cavity preparation. The adult mice were housed at 22 °C ± 2 °C temperature, 55% ± 5% humidity, and artificial illumination lit for 12 h with free access to food and water.

### 2.6 Pulp cavity exposure and drug delivery

Eight-week-old male mice were anesthetized with an intraperitoneal injection of avertin (250 mg/kg; T4802-5G, Sigma Aldrich). The pulp cavity on the upper right first molar was prepared using a 0.6 mm round bur and refined with K-files to minimize heat generation under a dissecting microscope. After pulp cavity exposure, mice were randomly assigned to two groups, and either 100 μM Thiamet-G or 1% dimethyl sulfoxide (DMSO) with pluronic® F-127 (P2443-250G, Sigma Aldrich) was locally delivered into the exposed pulp using a Hamilton syringe. Following drug delivery, the exposed pulp cavity was covered with mineral trioxide aggregate (MTA) and light-cured composite resin. The mice were then housed for 3, 5, and 42 days for further evaluation. 5–10 mice were used for each group.

### 2.7 Histology and immunohistochemistry

Mice were euthanized by cervical dislocation after 3, 5, and 42 days following local drug delivery; maxillae were separated, fixed in 4% paraformaldehyde (PFA), decalcified with 0.5% ethylenediaminetetraacetic acid (EDTA), dehydrated in graded ethanol (EtOH), cleared in xylene, and embedded in paraffin. Frontal wax sections were then prepared with a thickness of 7 μm using a microtome. At first, histomorphological alterations were analyzed by performing HE staining and MTC staining, as described previously (Aryal et al., 2021). For immunohistochemistry, anti-NESTIN (AB11306, Abcam), anti-TGF-β (ab92486, Abcam), anti-OPN (sc-73631, Santa Cruz Biotechnology), anti-MPO (bs-4943R, Bioss), anti-TNF-α (ab9739, Abcam), anti-NF-κB (bs-50467R, Bioss), anti-O-GlcNAc (RL2) (MA1-072, Invitrogen), and anti-RUNX2 (ab192256, Abcam) primary antibodies were used with goat anti-Mouse IgG H&L (HRP) (ab6789, Abcam) or goat F(ab')2 anti-Rabbit IgG F(ab')2 (HRP) (ab6112, Abcam) secondary antibodies. The color reaction was visualized using a diaminobenzidine tetrahydrochloride (DAB) reagent kit (C09-12, Origene). The experiment was conducted using at least three biological replicates. Then, the images of the immune-stained sections were arranged using Photoshop.

### 2.8 Micro-computed tomography (micro-CT) evaluation

After 6 weeks of Thiamet-G or DMSO treatment, mice were sacrificed by cervical dislocation, fixed in 4% PFA and maxillae were analyzed using micro-CT imaging (Skyscan1272; Bruker). Three-dimensional reconstructions were prepared using NRecon software to quantify the hard tissue formed in the region of interest as described previously (n = 3) (Aryal et al., 2021). *p* < 0.05 was considered statistically significant.

### 2.9 Photography and statistical analysis

All histological and immunostaining images were captured using a DM2500 microscope (Leica) equipped with a digital CCD camera (DF310 FX, Leica). Data are presented as mean ± SD, with at least three independent biological replicates performed for each experiment. Normality and homogeneity of variances were assessed prior to statistical analysis. Two-group comparisons were performed using the unpaired two-tailed Student’s t-test, while multiple group comparisons were analyzed by one-way ANOVA followed by Dunnett’s post hoc test. Statistical analyses were conducted using Microsoft Excel and GraphPad Prism 9 (GraphPad Software, USA). A *p*-value <0.05 was considered statistically significant.

## 3 Results

### 3.1 Thiamet-G treatment upregulates odontoblast differentiation markers *in vitro*

**FIGURE 1:**
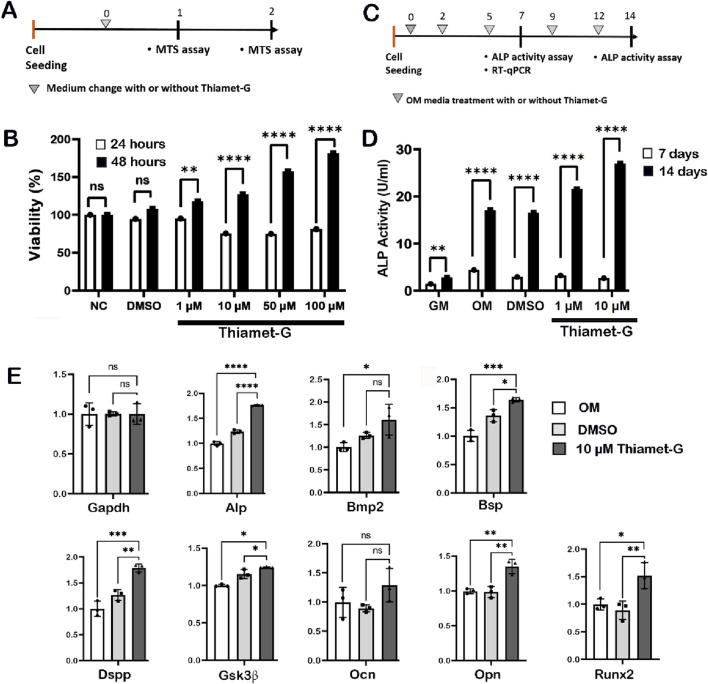
Effect of Thiamet-G on human dental pulp stem cells (hDPSCs). **(A)** Experimental design for MTS assay to examine cell viability and proliferation. **(B)** MTS assay shows that Thiamet-G treatment facilitates dose-dependent hDPSC proliferation. **(C)** Experimental design for ALP activity assay. **(D)** Thiamet-G treatment significantly induces ALP activity in hDPSCs compared to the control, particularly after 14 days **(E)** Thiamet-G treatment increases the expression of reparative dentin-related markers, including Alp, Bmp2, Bsp, Dspp, Opn, Gsk3β, and RUNX2, compared to the control. *Ns* non-significant, *p < 0.03, **p < 0.002, ***p < 0.0002, and ****p < 0.0001. *NC* negative control, *GM* growth medium, *OM* osteogenic medium.

To understand the potential role of Thiamet-G in dental pulp stem cell expansion and differentiation, we used *in vitro* cell cultivation using hDPSCs (Figure 1). An MTS assay was conducted to assess cytotoxicity and cell viability of the hDPSCs after treatment with various concentrations of Thiamet-G (1 μM, 10 μM, 50 μM, and 100 μM) (Figure 1A). The MTS assay results indicated that Thiamet-G exhibited no toxicity in hDPSCs and promoted dose-dependent cellular proliferation (Figure 1B). We then evaluated the ALP activity of hDPSCs treated with Thiamet-G (1 μM and 10 μM) for 7 and 14 days under osteogenic differentiation conditions (Figure 1C). After 7 days, the ALP activity was almost similar across all groups. However, after 14 days, the ALP activity significantly increased in Thiamet-G-treated groups in a dose-dependent manner, with 10 μM showing the highest activity (Figure 1D). Based on these, 10 μM was selected for further *in vitro* experiments. Then, we examined the global O-GlcNAc level using Western blot. Western blot data showed significantly increased global O-GlcNAc protein levels in Thiamet-G-treated groups compared to the control groups (Supplementary Figure S1). Thereafter, we examined the expression levels of reparative dentin formation markers, Alp, bone morphogenetic protein-2 (Bmp2), bone sialoprotein (Bsp), dentin sialophosphoprotein (Dspp), glycogen synthase kinase-3 beta (Gsk3β), osteocalcin (Ocn), osteopontin (Opn), and Runx2, after 7 days of Thiamet-G treatment with induced osteogenic differentiation, using RT-qPCR (Figure 1E). Our results showed significantly elevated expression patterns of all reparative dentin markers following Thiamet-G treatment. However, the mRNA expression of Ocn showed no significant changes compared to the control (Figure 1E).

### 3.2 Thiamet-G treatment facilitates reparative dentin formation *in vivo*

**FIGURE 2:**
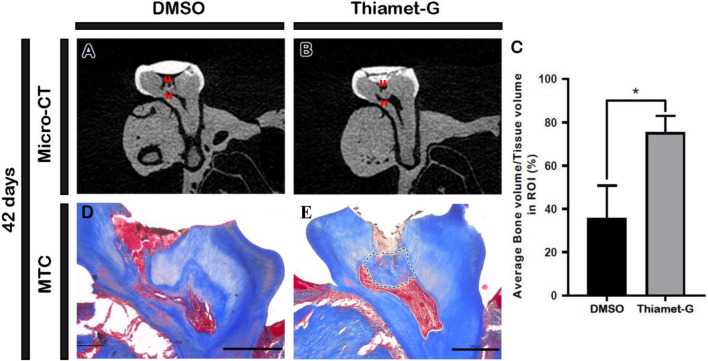
Thiamet-G treatment facilitates reparative dentin formation *in vivo*. **(A,B)** Micro-CT images show pulp cavity access prepared region and newly formed dentin-bridge after 42 days of local drug delivery (indicated by arrows). **(C)** Statistical analysis shows significantly higher hard tissue within the region of interest (ROI) in the Thiamet-G-treated specimens compared to the control (N = 3). **(D,E)** MTC staining after 42 days of local drug delivery following pulp cavity access preparation reveals a newly formed dentin-bridge (denoted with dashed lines) in Thiamet-G-treated specimens with sound pulp tissue. Scale bars: 200 μm **(C,D)**. *p < 0.03.

After 42 days of Thiamet-G treatment, dentin-bridge formation in the pulp cavity was evaluated using micro-CT and MTC staining. Micro-CT analysis revealed a significantly increased percentage of hard tissue volume in the Thiamet-G-treated specimens compared to the control, indicating facilitated reparative dentin formation (Figures 2A-C). MTC staining further showed that the Thiamet-G-treated group exhibited dentin-bridge formation beneath the pulp-exposed area, with regular pulp cell arrangement and well-organized tertiary dentin (Figure 2E). In contrast, specimens treated with DMSO showed disordered arrangement (Figure 2D). These results suggest that Thiamet-G treatment promotes effective reparative dentin formation with sound pulp tissue.

### 3.3 Thiamet-G treatment modulates inflammatory reactions during reparative dentin formation

**FIGURE 3:**
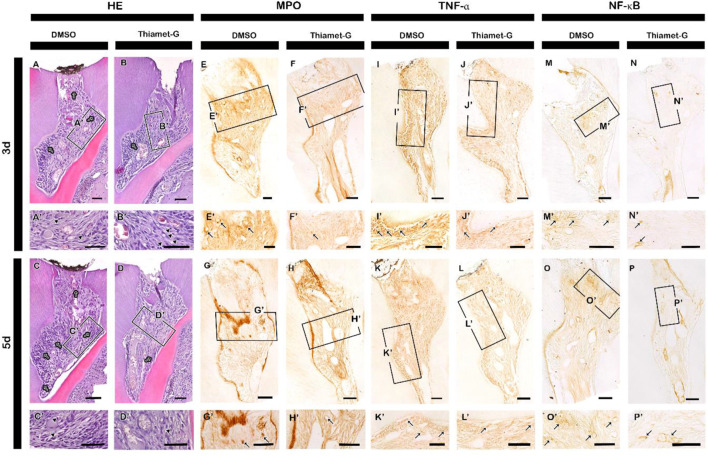
Thiamet-G treatment modulates inflammatory responses during reparative dentin formation. **(A–D’)** HE staining of pulp tissue following access cavity preparation at 3 and 5 days. At 3 days, leukocyte are increased in Thiamet-G–treated specimens compared with controls, whereas hyperchromatic cells are more abundant in control specimens than in Thiamet-G treated specimens. At 5 days, control specimens exhibit higher leukocyte numbers and increased hyperchromatic cells compared with Thiamet-G–treated specimens. Arrowheads indicate leukocytes, and arrows denote hyperchromatic cells. **(E–P’)** The localization of inflammation-related proteins, such as MPO, TNF-α, and NF-κB, is decreased in Thiamet-G-treated specimens compared to the controls. (E-P’) Arrows indicate positive reactions of the respective proteins. Scale bar: 50 μm.

As Thiamet-G treatment for 42 days promoted reparative dentin formation, we examined the early cellular and molecular responses of pulp cells against Thiamet-G treatment using histology and immunohistochemistry after 3 and 5 days of local drug delivery (Figure 3). Following 3 and 5 days of local drug delivery, histological alterations were examined using HE staining. Thiamet-G treatment produced time-dependent alterations in inflammatory cell presence. After 3 days of treatment, histological analysis showed a significant increase in leukocyte infiltration, whereas the number of hyperchromatic cells was reduced compared with controls (Figures 3A–B’; Supplementary Figure S2). In contrast, following 5 days of Thiamet-G exposure, both leukocyte numbers and hyperchromatic cell counts were significantly decreased (Figures 3C–D’; Supplementary Figure S2). These data indicate that Thiamet-G modulates inflammatory responses in a time-dependent manner, characterized by early immune cell accumulation followed by attenuation of inflammation with prolonged treatment. Thiamet-G-treated specimens exhibited a higher number of O-GlcNAc-positive cells at 3 days post-treatment compared to controls (Supplementary Figures S3A,B). Although the number decreased by day 5, it remained elevated relative to control levels particularly beneath injury area (Supplementary Figures S3C,D). After 3 and 5 days of Thiamet-G local delivery, we examined the role of Thiamet-G treatment in inflammation modulation by performing immunohistochemistry with its well-known markers MPO and TNF-α. At 3 days of Thiamet-G local delivery, decreased immunoreactions against MPO and TNF-α were observed compared to control (Figures 3E,F’,I–J’; Supplementary Figure S4). Similarly, Thiamet-G treatment for 5 days showed decreased localization of MPO and TNF-α compared to control specimens (Figures 3G,H’,K–L’; Supplementary Figure S4). Furthermore, localization of NF-κB, the marker for innate and adaptive immune responses, showed a decreased reaction after 3 and 5 days of Thiamet-G treatment compared to control (Figures 3M–P’; Supplementary Figure S4).

### 3.4 Thiamet-G treatment promotes the localization of reparative dentin-related proteins

**FIGURE 4:**
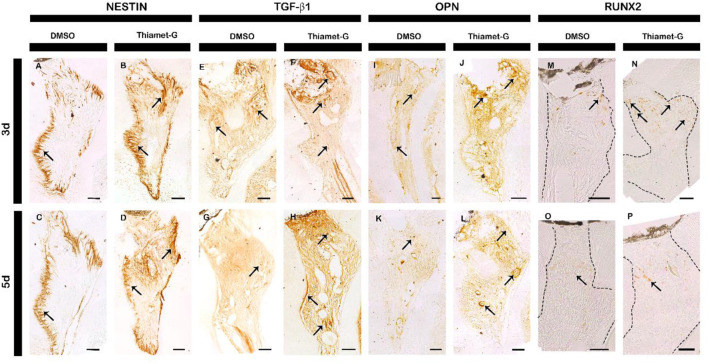
Thiamet-G treatment facilitates reparative dentin-related protein localization. Thiamet-G-treated specimens for 3 and 5 days show intense immunostaining for **(A–D)** NESTIN, **(E–H)** TGF-β1, and **(I–L)** OPN compared to control. Furthermore, **(M–P)** the number of RUNX2-positive cells is also significantly higher in Thiamet-G-treated specimens for 3 and 5 days compared to the control (indicated by arrows). Dashed lines indicate pulp tissues and arrows indicate positive reaction of respective proteins. Scale bar: 50 μm.

Further, we examine the localization patterns of NESTIN, TGF-β1, OPN, and RUNX2 in the *in vivo* experimental specimens as markers of reparative dentin formation. Immunohistochemistry using NESTIN antibody showed a stronger staining pattern, particularly around the injury area and reactionary dentin-forming region of Thiamet-G treated specimens for 3 and 5 days compared to the control (Figures 4A–D; Supplementary Figure S4). At 3 days of Thiamet-G treatment, the localization of TGF-β1, one of the markers of dental pulp tissue repair, was comparatively similar to that of control (Figures 4E,F; Supplementary Figure S4). However, Thiamet-G-treated specimens for 5 days showed more intense immunostaining against TGF-β1 than the control (Figures 4G,H; Supplementary Figure S4). Moreover, immunostaining against OPN, one of the markers for reparative dentin formation, also showed intense staining, particularly beneath the injury site in the specimens treated with Thiamet-G for 3 days and 5 days compared to the control (Figures 4I–L; Supplementary Figure S4). At 3 days of local delivery, more RUNX2-positive cells were observed beneath the injury area of Thiamet-G-treated specimens than in controls (Figures 4M,N; Supplementary Figure S4). Although the number of RUNX2-positive cells was less in Thiamet-G-treated specimens for 5 days compared to 3 days, it was still more than that of control specimens (Figures 4O,P; Supplementary Figure S4). Notably, a significant increase in RUNX2-positive cells was observed in the exposed pulp.

## 4 Discussion

In mild dentin and enamel damage, odontoblasts secrete reactionary dentin, while pulp exposure stimulates stem-cell driven reparative dentin formation (Neves and Sharpe, 2018; Njeh et al., 2016). However, this natural process of dentin regeneration is slow and often not sufficient to preserve dental-pulp properly. Unlike embryonic dentinogenesis, the mechanisms regulating post-natal dentin repair are poorly understood. This study used Thiamet-G, an inhibitor of OGA, a positive regulator of osteoblast differentiation and anti-inflammatory agent, which acutely augments global O-GlcNAcylation (Nagel and Ball, 2014; Koyama and Kamemura, 2015; Li et al., 2022; Hilgers et al., 2012). Due to the perinatal lethality associated with the genetic knockout of OGA (Yang et al., 2012) and the limitations of other inhibitors, such as PUGNAc, which has off-target effects on other glycosidases, Thiamet-G was chosen to examine how O-GlcNAcylation influences pulp cell behavior and reparative dentin formation during tooth injury.

Several recent studies linked the regulatory coordination actions of OGT and OGA during immune cell development, homeostasis, and functions (Chang et al., 2020). Inflammatory responses are primary and essential mechanisms during tissue repair and regeneration for restoring homeostatic states after tissue injury (Eming et al., 2017). Unresolved inflammation leads to disease progression or complete tissue damage by inhibiting tissue repair processes (Cooper et al., 2014). Following inflammation after caries or trauma, primarily, immune cells, such as neutrophils and macrophages, are produced within the pulp to modulate different aspects of the inflammatory responses until eradicating the injurious agent from the injury area (Galler et al., 2021; Liu et al., 2016; Su et al., 2019; Aratani, 2018). Therefore, controlled inflammatory responses are crucial to dentin-pulp repair and regeneration (Aryal et al., 2021). In our study, control specimens exhibited stronger immunostaining for MPO and TNF-α, indicating that inflammatory responses were still active. In contrast, the decreased localization of MPO and TNF-α in Thiamet-G-treated samples highlights its role in modulating inflammation during the early stages of dentin-pulp repair. Consistent with our findings, several studies have revealed that Thiamet-G treatment could reduce the presence of activated microglia/macrophages at injury sites (Hilgers et al., 2012; Yao et al., 2018). A previous study demonstrated that Thiamet-G treatment reduced inflammation in a mouse stroke model by modulating microglia/macrophage phenotype and inhibiting NF-κB p65 signaling, thereby suppressing the immune response following ischemic injury (He et al., 2017). Similar to this finding, NF-κB localization was also reduced in Thiamet-G-treated specimens (Figures 4N,P). These findings suggest that Thiamet-G treatment could modulate inflammation during the early stage of dentin-pulp repair.

Injury or trauma would lead to infection and inflammation of the pulp tissue as an initial response, with tissue repair commencing after the inflammation is resolved (Cooper et al., 2014; Gaudin et al., 2015), followed by stem/progenitor cell proliferation, differentiation, and mineralization (Cooper et al., 2014). Consequently these stem/progenitor cells would secrete reparative dentin, resulting in dentin bridge formation above the exposed pulp, thus facilitating the regeneration of pulp tissue in the injury site (Cooper et al., 2014; Yu and Abbott, 2016). In our study, Thiamet-G treatment increased hDPSC proliferation in a dose-dependent manner (Figure 2B). This result suggests that Thiamet-G treatment could potentially enhance the initial expansion of pulp cells during dental pulp repair, thereby promoting dentin secretion. However, studies on various other cell types, including preosteoblasts, chondroblasts, BMSCs, and osteoblasts, demonstrated no effect of Thiamet-G on their proliferation (Li et al., 2022; Yan et al., 2024; Nagel and Ball, 2014; Zhang et al., 2023). Furthermore, increased ALP activity of hDPSCs treated with Thiamet-G indicated active mineralization, while increased expression of genes, such as Dspp, Bmp2, Bsp, and Runx2, suggested facilitated odontoblastic differentiation, as previously reported (Figure 1) (Hilgers et al., 2012; Nagel and Ball, 2014; Sun et al., 2019; Hu et al., 2022). Localization of the active odontoblast marker, NESTIN was notably increased in Thiamet-G-treated specimens, particularly beneath the injury site (Figure 4) . Thiamet-G treatment also promoted the expression and localization patterns of reparative dentin-related molecules such as RUNX2. RUNX2, a key transcriptional factor involved in osteoblast and odontoblast differentiation by regulating genes such as OCN, BSP, and DSPP (Han et al., 2014; Vijaykumar et al., 2020). It is also essential for matrix formation, remodeling, and hard tissue mineralization (Sun et al., 2019), and it’s O-GlcNAcylation has been reported to regulate genes, such as Alp, Ocn, and β-catenin, during osteogenesis (Sun et al., 2019; Andrés-Bergós et al., 2012; Nagel and Ball, 2014). Notably, increased localization of RUNX2 and OPN was observed beneath the injury area in the course of reparative dentin formation in this study. Previous study revealed that OPN deficiency impairs dentin regeneration suggesting its role (Saito et al., 2016) (Figure 4). The newly formed dentin bridge with sound pulp in Thiamet-G-treated specimens suggests its potential function for dental pulp repair and dentin regeneration by simultaneously attenuating excessive inflammation in the pulp and enhancing odontoblast-like differentiation and mineralized matrix deposition, rather than acting through a single pathway alone. To elucidate the detailed molecular mechanisms underlying these effects, we are currently conducting a separate project using an *in vitro* study model.

In conclusion, our findings suggest Thiamet-G as a potential therapeutic agent for dentin-pulp repair. Particularly, by incorporating Thiamet-G into restorative materials such as Mineral Trioxide Aggregate for pulp coverage might enhance its efficacy by modulating inflammation and activating signaling pathways associated with reparative dentin formation. This approach offers targeted and minimally invasive strategy for pulp regeneration.

## Data Availability

The original contributions presented in the study are included in the article/Supplementary Material, further inquiries can be directed to the corresponding authors.
