# Supplementary material for: Thiamet-G facilitates reparative dentin formation via modulating O-GlcNAcylation and inflammation
Source: Front Physiol. 2026 Jan 16;16:1739168. doi: 10.3389/fphys.2025.1739168 (PMC12855040; doi:10.3389/fphys.2025.1739168)
Supplement: Supplementary file 1 [file DataSheet1.docx]

**Thiamet-G facilitates reparative dentin formation via modulating O-GlcNAcylation and inflammation**

**Supplementary data**

**Method**

**Western blot**

After 7 and 14 days of hDPSC *in vitro* cultivation with differentiation media, cell lysates were prepared using radioimmunoprecipitation assay (RIPA) lysis and extraction buffer (89900, ThermoFisher Scientific) with protease inhibitor cocktail (A32953, ThermoFisher Scientific). The protein concentration of the cell lysate was measured using a BCA assay, following the manufacturer’s instructions. Then, an equal amount of protein was run in Mini-Protean TGX Stain-Free Precast Gels (4568104, BIO-RAD). Proteins were transferred onto Trans-Blot Turbo Mini 0.2 μm PVDF Transfer Packs (1704156, BIO-RAD). The transferred blots were incubated with 5% bovine serum albumin in tris-buffered saline containing 0.01% Tween-20 (TBST) to block unspecific binding of the antibodies. The transferred proteins were incubated overnight with anti- O-GlcNAc (CTD110.6) primary antibody (9875, Cell Signaling Technology) at 4 °C. Next day, blots were washed with TBST and incubated with horseradish peroxidase (HRP)-conjugated secondary antibody for an hour at RT. Finally, positive signals were detected using SuperSignal™ West Femto Maximum Sensitivity Substrate (34095, ThermoFisher Scientific). The band intensity of target protein (O-GlcNAc) was measured using ImageJ software, and background intensity was subtracted. The relative protein expression levels were normalized to β-actin as a loading control. Data are presented as fold change relative to the control group.

**Statistical analysis**

Leukocytes were identified by an experienced pathologist from H&E-stained sections and quantified within a 100 × 100 μm² area. For the quantification of immunohistochemistry, RUNX2- and O-GlcNAc–positive cells were counted within 50 × 50 μm² regions from at least five sections per group derived from three independent experiments. For other markers (MPO, TNF-α, NF-κB, NESTIN, TGF-β1, OPN), staining intensity was quantified as the mean gray value (average pixel intensity) within 50 × 50 μm² regions using ImageJ software. Background intensity from tissue-free areas was subtracted to obtain corrected mean gray values. Statistical comparisons were performed using two-way ANOVA followed by Sidak’s multiple comparisons test (GraphPad Prism version 8). A *p*-value ≤ 0.033 was considered statistically significant.

**Supplementary table 1.** List of primers used in the study

| Genes | Accession No. | Primer sequence | | Size (bp) |
| --- | --- | --- | --- | --- |
| Gapdh | NM_001357943.2 | Forward | GTCTCCTCTGACTTCAACAGCG | 120 |
|  |  | Reverse | ACCACCCTGTTGCTGTAGCCA A |  |
| Alp | NM_001127501.4 | Forward | CTGCCATCCTGTATGGCAATG | 103 |
|  |  | Reverse | AGACTGCGCCTGTAGTTGTTG |  |
| Bmp2 | NM_001200.4 | Forward | TTTGGACACCAGGTTGGTGAA | 108 |
|  |  | Reverse | ACGAATCCATGGTTGGCGT |  |
| Bsp | NM_004967.4 | Forward | CAAGGGCACCTCGAAGACAA | 108 |
|  |  | Reverse | ACGGTGGTGGTTTTCCCAAA |  |
| Dspp | NM_014208.3 | Forward | CCGCATCTTCTTTTGCGT | 120 |
|  |  | Reverse | CAACCATAGAGAAAGCAAACGCG |  |
| Gsk3β | NM_001146156.2 | Forward | GGCAATTGCACTGTGTAGCC | 144 |
|  |  | Reverse | AAGAGTGCAGGTGTGTCTCG |  |
| Osteocalcin | NM_199173.6 | Forward | TAGTGAAGAGACCCAGGCGCTA | 109 |
|  |  | Reverse | TCACAGTCCGGATTGAGCTCA |  |
| Osteopontin | NM_001040060.2 | Forward | GTGGGAAGGACAGTTATGAA | 151 |
|  |  | Reverse | CTGACTTTGGAAAGTTCCTG |  |
| Runx2 | NM_004348 | Forward | CCCAGTATGAGAGTAGGTGTCC | 149 |
|  |  | Reverse | GGGTAAGACTGGTCATAGGACC |  |


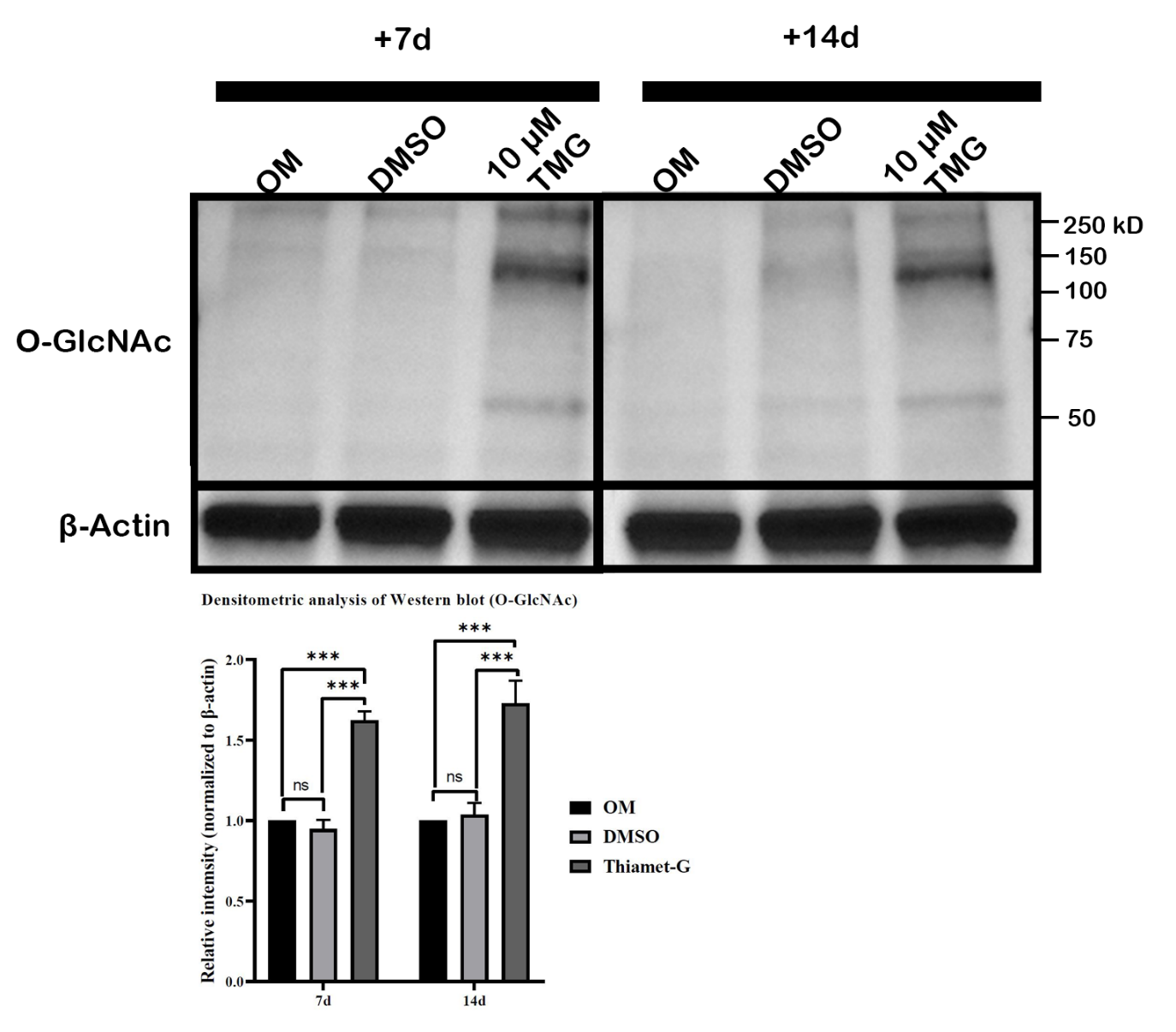


**Supplementary figure 1 (S1). Western blot analysis of O-GlcNAc.** The total protein levels of O-GlcNAc (CTD110.6) were increased in Thiamet-G-treated hDPSCs after 7 and 14 days of osteogenic differentiation, compared to both the negative control (OM: Osteogenic medium) and vehicle control (DMSO) groups. Statistical significance is indicated as **p* < 0.033, ****p* < 0.001; ns, not significant.


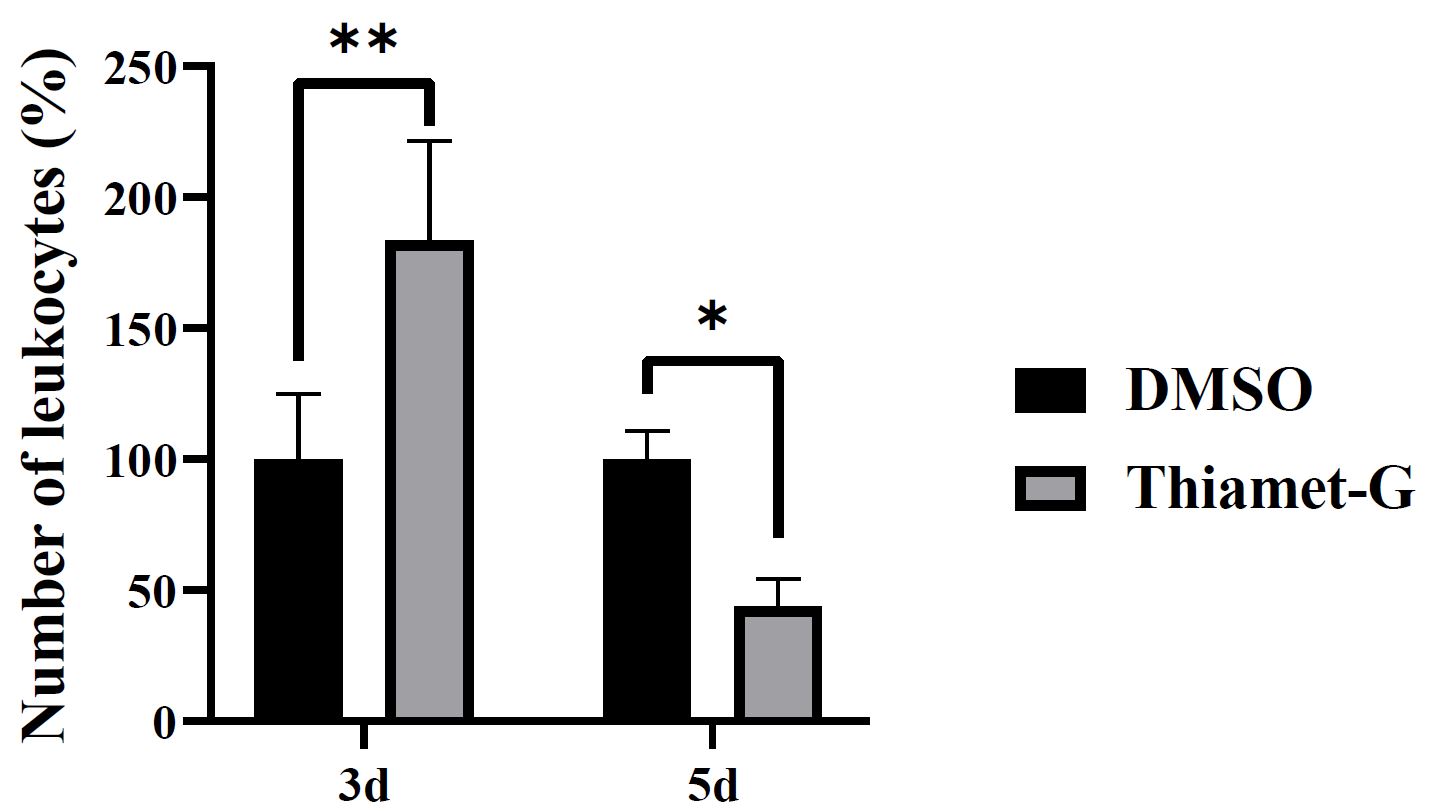


**Supplementary figure 2 (S2). Time-dependent effect of Thiamet-G on leukocyte numbers**

Leukocyte counts at 3 and 5 days following Thiamet-G treatment compared with DMSO control. Thiamet-G increased leukocyte numbers at 3 days but significantly reduced them at 5 days. Data are presented as mean ± SD. Statistical significance is indicated as ***p* < 0.02, ****p* < 0.001.

**
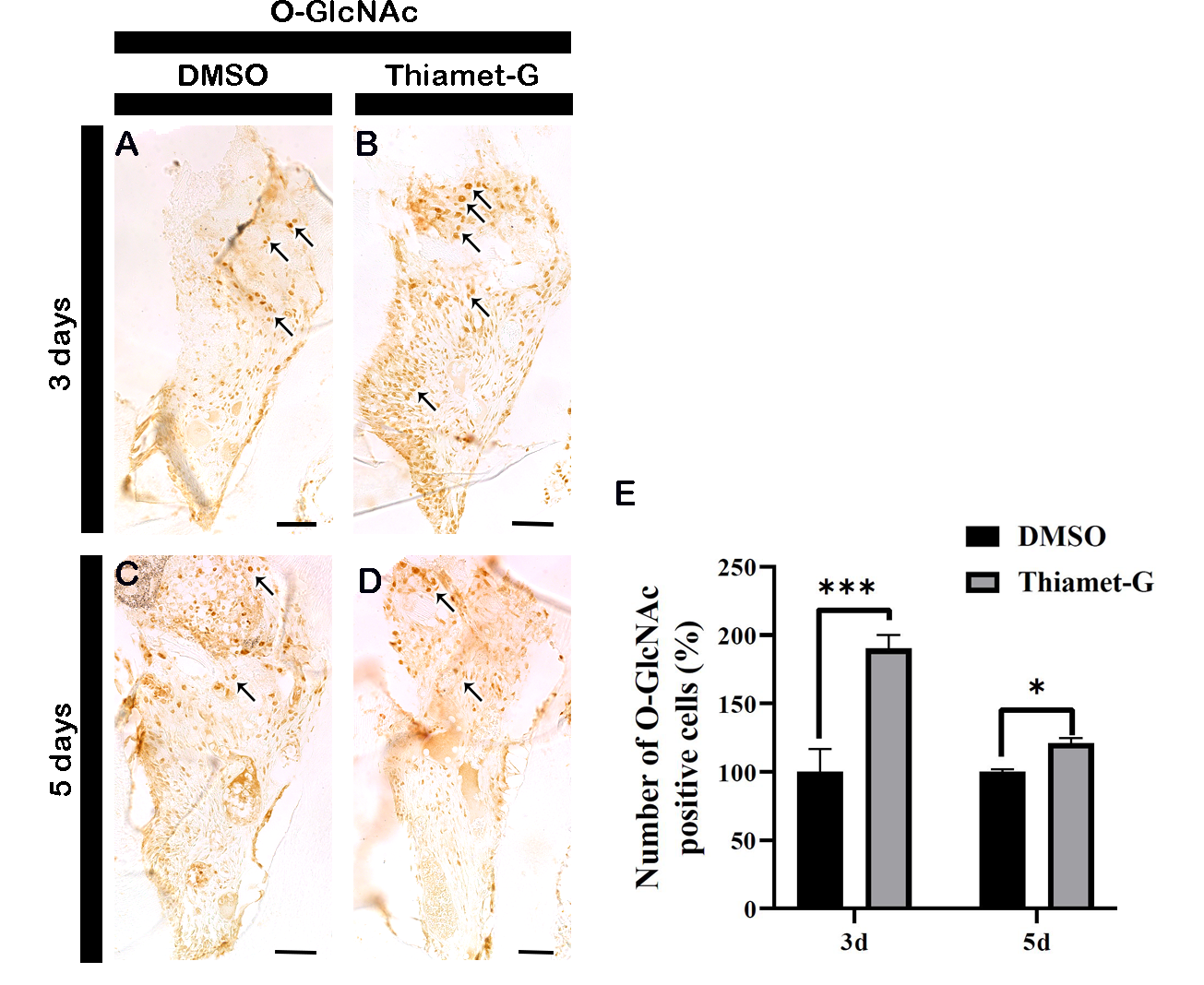
**

**Supplementary figure 3 (S3). Immunohistochemistry analysis of O-GlcNAc (RL2). (B)** Thiamet-G-treated specimens showed a higher number of O-GlcNAc-positive cells compared to the control group **(A)** at 3 days after local drug delivery. Although the number of O-GlcNAc-positive cells decreased in Thiamet-G-treated specimens at 5 days **(D)**, it remained higher than in the control group, particularly beneath the injury site **(B). (E)** Quantitative analysis of O-GlcNAc–positive cells. Arrows denotes O-GlcNAc positive cells. Scale bars: 50 μM. Statistical significance is indicated as **p* < 0.03 and ****p* < 0.001.

**
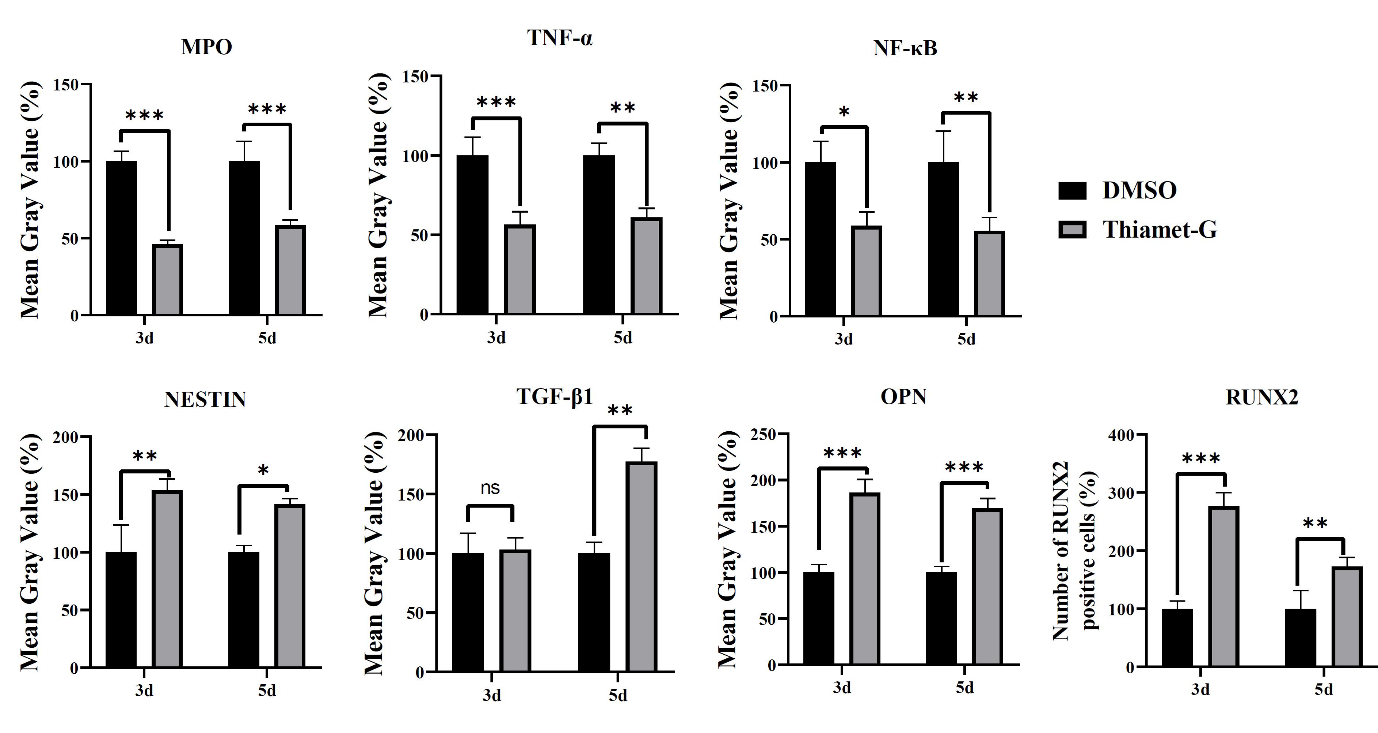
 Supplementary figure 4 (S4). Quantitative analysis of immunohistochemical staining.** Bar graphs show the quantification of MPO, TNF-α, NF-κB, NESTIN, TGF-β1, OPN (mean gray value, %), and RUNX2 (number of positive cells) in DMSO and Thiamet-G treated groups at 3 and 5 days after treatment. Data are presented as mean ± SD. Statistical significance is indicated as **p* < 0.03, ***p* < 0.02, ****p* < 0.001; ns, not significant.
